# miRNA expression profiling of Epstein–Barr virus‐associated NKTL cell lines by Illumina deep sequencing

**DOI:** 10.1002/2211-5463.12027

**Published:** 2016-02-27

**Authors:** Julia Alles, Jennifer Menegatti, Natalie Motsch, Martin Hart, Norbert Eichner, Richard Reinhardt, Gunter Meister, Friedrich A. Grässer

**Affiliations:** ^1^ Institute of VirologySaarland University Medical SchoolHomburg/SaarGermany; ^2^ Biochemistry Center Regensburg (BZR)Laboratory for RNA BiologyUniversity of RegensburgRegensburgGermany; ^3^ Max Planck Genome Centre CologneCologneGermany; ^4^ Present address: Boehringer IngelheimBirkendorfer Strasse 65D‐88397BiberachGermany

**Keywords:** CUL5, ebv‐miR‐BART16, Epstein‐Barr virus, hsa‐miR‐148a, microRNA, S1PR1

## Abstract

The aim of this work was to establish the microRNA profile of SNK6 and SNT16, two Epstein–Barr virus (EBV)‐infected cell lines derived from nasal NK/T‐cell lymphoma (NKTL). The oncogenic EBV is strongly associated with the pathogenesis of nasal and extranodal NK/T‐cell lymphoma and expresses 44 mature microRNAs and two noncoding EBV‐encoded RNAs (EBERs). miRNAs are 19‐25nt noncoding RNAs that affect host and viral gene expression post‐transcriptionally. Deregulated miRNA patterns are frequently linked to a variety of human cancers including lymphomas. miRNA profiling of the two NK/T cell lines vs. primary cells revealed 10 and 4 up‐regulated and 10 and 12 down‐regulated miRNAs in SNK6 and SNT16 cells respectively. The results were validated by qRT‐PCR for selected miRNAs. Target gene analyses confirmed cullin 5 (CUL5) and sphingosin‐1‐phosphate receptor 1 (S1PR1) as targets for the down‐regulated hsa‐miR‐148a and viral ebv‐miR‐BART16 respectively. As recently demonstrated for the regulation of IL1‐alpha by miR‐142‐3p, coexpression of the EBERs selectively exerted corepression of S1PR1 by BART16 but not of CUL5 by miR‐148a, indicating selective corepression by the EBERs.

**Abbreviations**
BL: Burkitt's lymphoma
DLBCL: diffuse large B‐cell lymphoma
EBER: Epstein–Barr virus‐encoded RNA
EBV: Epstein–Barr virus
GC: gastric carcinoma
GI: gastrointestinal
HL: Hodgkin lymphoma
LCL: lymphoblastoid cell line
miR‐/miRNA: microRNA
mut: mutated
NKTL: NK/T‐cell lymphoma
NPC: nasopharynx carcinoma
nt: nucleotide
PTLD: post‐transplant lymphoproliferative disease
RISC: RNA‐induced silencing complex
wt: wild‐type

The Epstein–Barr virus (EBV) infects approximately 95% of the adult human population worldwide and generally establishes a symptomless, latent infection 1. Under certain conditions, however, it induces malignant tumours of B‐ or T‐cell origin, including Burkitt's lymphoma (BL), Hodgkin's lymphoma (HL), as well as epithelial tumours such as nasopharyngeal carcinoma (NPC) and gastric carcinoma (GC) 2. The tumorigenic potential is reflected in its ability to readily transform B‐cells into permanently growing cell lines (lymphoblastoid cell lines, LCLs), the *in vitro* equivalent of post‐transplant lymphoproliferative disease (PTLD) that can arise in immunosuppressed patients 3. EBV is also strongly associated with a type of non‐Hodgkin's Lymphoma: the very rare NK/T‐cell lymphoma (NKTL). Those tumours derive from NK‐ and/or T cells 4 and occur predominantly in Asia and Central‐/South America. For nasal NKTL the upper aerodigestive tract is often affected by a high grade of necrosis 5 as a consequence of perforin 6 or granzyme B expression 7. Cases of extranodal NKTL of the gastro‐intestinal tract, skin, testis, lung, eye or soft tissue have also been reported 8, 9, 10, 11, 12).

In addition to protein‐encoding genes, EBV was the first virus where microRNAs (miRNAs) were described 13 and these play important roles in transformation by EBV 14, 15, 16. MiRNAs are conserved, small noncoding RNAs of approximately 22 nt length. They repress gene expression through binding to partially complementary sequences usually located in the 3′ untranslated region (UTR) of target mRNAs 17. To carry out their regulatory functions, miRNAs are incorporated into RNA‐induced silencing complexes (RISC) where they directly interact with a member of the Argonaute (Ago) protein family 18. Upon binding to their distinct target sites, miRNA‐Argonaute complexes recruit a member of the glycine‐tryptophan‐rich motif (GW) protein family, which recruits the deadenylation machinery leading to poly(A) tail shortening and finally mRNA decay. At early stages of repression, however, the GW protein coordinates translational repression of the mRNA without considerably affecting mRNA stability 19, 20, 21. Like cellular miRNAs, viral miRNAs can be secreted in endosome‐derived exosomes, and these show an enrichment for 3′ end uridinylated isoforms 22. RIS complexes are associated with endosomal membranes 23, 24 and knock down of GW182 reduces exosomal miRNA secretion 25, linking the mechanisms of miRNA activity and release. EBV also encodes two non‐polyadenylated RNAs (Epstein‐Barr virus Encoded RNA; EBER) 26. These are transcribed by RNA polymerase III and are of 167 (EBER1) and 172 (EBER2) nucleotides (nt) length. The EBER transcripts are expressed at up to 10^6^ copies per cell in all EBV‐transformed tumours and cell lines 26, 27. We have recently shown the EBERs exert an additional, miRNA‐specific down‐regulation on miRNA targets: co‐expression of the EBERs further down‐regulated the protein expression of interleukin 1α and RAC1 reporters by miR‐142‐3p and also the protein expression of interleukin 1α, but not of ADCY9, another known target of miR‐142‐3p 28. Further, the EBERs had no effect on the down‐regulation of TOMM22, a known target for the EBV‐encoded miRNA ebv‐miR‐BART16 29.

Due to its high aggressiveness and accompanying necrosis, the amount of primary tumour tissues available is very limited. We therefore chose to compare normal CD56+/CD3+ cells from healthy donors with the NKTL lines SNK6 and SNT16. Furthermore, we validated new targets for viral and deregulated host miRNAs. For the sphingosin‐1‐phosphate receptor 1 (S1PR1), we find that co‐expression of the EBERs further represses down‐regulation of both a 3′UTR reporter and the protein by ebv‐miR‐BART16, while the EBERs show no additional effect on the repression of a 3′UTR cullin‐5 (CUL5) reporter by miR‐148a.

## Materials and methods

### Cell culture

All cell lines were cultured as described previously 30, 31, 32, 33. The LCL cell lines AM 29 and AM 58 established with a complete and an EBER‐deleted EBV genome 34 were a generous gift from Sankar Swaminathan, University of Utah, Salt Lake City, Utah, USA. SNK6 35 and SNT16 36 cells were a kind gift from Martin Rowe, University of Birmingham, Birmingham, UK.

### Isolation of CD56+/CD3+ primary cells

PBMC were isolated from buffy coats (Blutspendezentrale Saar‐Pfalz GmbH, Homburg, Germany) by Ficoll separation. CD56+/CD3+ cells were isolated from PBMC using the human CD56+/CD3+ NKT MACS cell isolation kit according to the manufacturer's instructions (Miltenyi Biotec, Bergisch Gladbach, Germany).

### RNA sequencing

RNA‐Seq libraries were prepared according to supplier recommendations (TruSeq DNA/RNA sample preparation v2 guide: https://www.neb.com/protocols/1/01/01/library-preparation-e7300). Libraries were quantified by fluorometry, immobilized and processed onto a flow cell with a cBot followed by sequencing as a 100 bp single read using TruSeq v3 chemistry on HiSeq2500 (all components by Illumina, San Diego, CA, USA). Data analysis was performed using in‐house written scripts. In short, after initial quality check and adapter‐trimming the remaining valid reads were mapped against human and EBV miRNAs listed in mirbase version 20 (June 2013; www.mirbase.org). The minimum length of annotated reads was set to 18nt and no mismatches were allowed. Annotated miRNA reads were reviewed for multiple insert annotations and then normalized to the number of valid reads in the corresponding library. Finally, the normalized values of SNK6 and SNT16 cell lines were compared to CD56+/CD3+ to calculate fold changes.

### Reverse transcription and quantitative real‐time PCR

DNase I‐treated RNA was reverse transcribed using the miScript II RT Kit (Qiagen, Hilden, Germany). Semi‐quantitative RT‐PCR was conducted with the LightCycler 1.5 System (Roche Diagnostics, Mannheim, Germany). miRNAs were amplified using the LightCycler^®^ FastStart DNAMaster^PLUS^ SYBR Green I Kit and reverse Primer 5′‐GCG AGC ACA GAA TTA ATA CGA C‐3′ with miRNA‐specific forward primers: qRT‐miR‐21‐5p: 5′‐TAG CTT ATC AGA CTG ATG TTG A‐3′, qRT‐miR‐148a‐3p: 5′‐TCA GTG CAC TAC AGA ACT TTG T‐3′, qRT‐miR‐150‐5p: 5′‐TCT CCC AAC CCT TGT ACC AGT G‐3′, qRT‐miR‐155‐5p: 5′‐TTA ATG CTA ATC GTG ATA GGG GTA A‐3′. For relative quantification, the ‘5.8sRNA'‐primer: 5′‐CTA CGC CTG TCT GAG CGT CGC TT‐3′ was used 32.

### Dual‐luciferase assays

Dual‐luciferase assays employing 3′UTR reporters in pMIR‐RNLTK (a dual firefly and renilla luciferase vector) were carried out in HEK293T cells as described 33. Typically, 10^5^ HEK293T cells were seeded in 24‐well format and transfected using PolyFect (Qiagen) with 0.2 μg·well^−1^ reporter vector and 0.8 μg effector plasmid. The ratio of firefly (reporter)/renilla (control) luciferase for each sample was determined (%RLU) and assays conducted in duplicate. Statistical significance was tested using Student's *t*‐test.

### Plasmids

The ebv‐miR‐BART16 and EBER expression plasmids had been described previously 28, 29, 37. The miR‐148a precursor was cloned into pSG5 using the following oligonucleotides: 5′‐EcoRI‐miR‐148a: 5′‐CGG AAT TCT GTT GGG CAT TTG CTC CTG C‐3′, 3′‐BamHI‐miR‐148a: 5′‐CGC GGA TCC CAG TCA GGA GTC CAC CAG GG‐3′. The 3′UTRs of S1PR1 (NM_001400.4) and CUL5 (NM_003478.3) were cloned into the modified dual‐luciferase reporter vector pMIR‐RNLTK 38 using the following oligonucleotides: 5′‐SpeI‐S1PR1: 5′‐GAC TAG TCG CAG CAA ATC GGA CAA TTC‐3′, 3′‐SacI‐S1PR1: 5′‐CGA GCT CGC TGG ACT GAA AAG TTT CTG GCG‐3′, 5′‐SpeI‐CUL5: 5′‐GGA CTA GTG TAA TGC TCA GCT GCA GAC‐3′, 3′‐SacI‐CUL5: 5′‐CGA GCT CCT GCA ATC ATA ATG ACC TAC C‐3. Mutation of miRNA binding sites within the 3′UTRs was carried out by overlap extension PCR and the following oligonucleotides: 5′ SspI‐SalI‐S1PR1mutBART16: 5′‐GCA TAA GGA AGA ATA TTG TCG ACA AAT GAT ATT ATG CC‐3′, 3′ SspI‐SalI‐S1PR1mutBART16: 5′‐GGC ATA ATA TCA TTT GTC GAC AAT ATT CTT CCT TAT GC‐3′, 5′ PmlI‐CUL5mut148a: 5′‐GAT CTT CAG ATA TTC ACA CGT GCA AAA AAT GCT GTT ATC‐3′, 3′ PmlI‐CUL5mut148a: 5′‐GAT AAC AGC ATT TTT TGC ACG TGT GAA TAT CTG AAG ATC‐3′.

### Western blotting

HEK293T cells were transfected with 2 μg plasmid(s) 24 h after seeding 4 × 10^5^ cells in 6‐well dishes. Cells were harvested 48 h later. Protein extracts (30 μg) were separated by 8,75% (CUL5) and 12.5% (S1PR1) PAGE and transferred to Protran^™^ membranes (Roth, Karlsruhe, Germany). After blocking, membranes were incubated with the following primary antibodies: anti‐human‐EDG1 (S1PR1) clone H‐60 (Santa Cruz), anti‐human‐CUL5 clone H‐300 (Santa Cruz, Heidelberg, Germany), anti‐human‐β‐actin clone 14C10 (Sigma‐Aldrich, Munich, Germany) and anti‐human‐GAPDH (Cell Signalling, Leiden, the Netherlands). After incubation with secondary antibodies coupled to horseradish peroxidase (Sigma‐Aldrich), the membranes were developed using ECL (Life Technologies, Braunschweig, Germany).

### Northern blotting

Total RNA from transfected HEK293T cells was extracted using peqGOLD Trifast (Peqlab, Erlangen, Germany) according to the manufacturer's description without washing steps. 20 μg RNA was separated on a 12% urea‐polyacrylamid gel and subsequently blotted onto a nylon membrane (Amersham, Freiburg, Germany) followed by chemical cross‐linking 28, 39. After hybridization with UT^32^P‐labelled antisense probes (miRVana probe construction kit; Thermo Fisher (Ambion), Braunschweig, Germany) overnight and washing steps, the blots were exposed to phosphor screens and developed using a Typhoon Scanner (Amersham).

## Results

### miRNA profiling of primary CD56+/CD3+ cells vs. SNK6/SNT16 cell lines

We had previously compared the miRNA profiles of primary nasal NK/T‐cell lymphoma (NKTL) with EBV‐negative T‐cell lymphoma and normal thymus 33. Since CD56+/CD3+ primary cells are the most likely precursor cells for NKTL 40, we established in the present study the miRNA profile of CD56+/CD3+ cells from healthy donors in comparison with the EBV‐positive NKTL lines SNK6 and SNT16. From a total of 5 393 355 valid sequences obtained from the CD56+/CD3+ cells, 985 467 reads (18.27%) could be assigned to human miRNAs, with only 54 reads obtained from viral sequences. Of the 5 674 444 reads from the SNT16 cell line, 1 201 368 reads (21.17%) represented human and 549 121 reads (9.68%) were from EBV miRNAs. For the SNK6 cell line, we obtained 2 327 119 valid sequences with 931 115 reads from human miRNAs (40.01%) and 160 749 EBV miRNA reads (6.91%). Viral miRNAs thus represented 31.37% and 14.72% of the total miRNA count in the SNK6 and SNT16 cells respectively (Tables S1 and S2).

**Figure 1:**
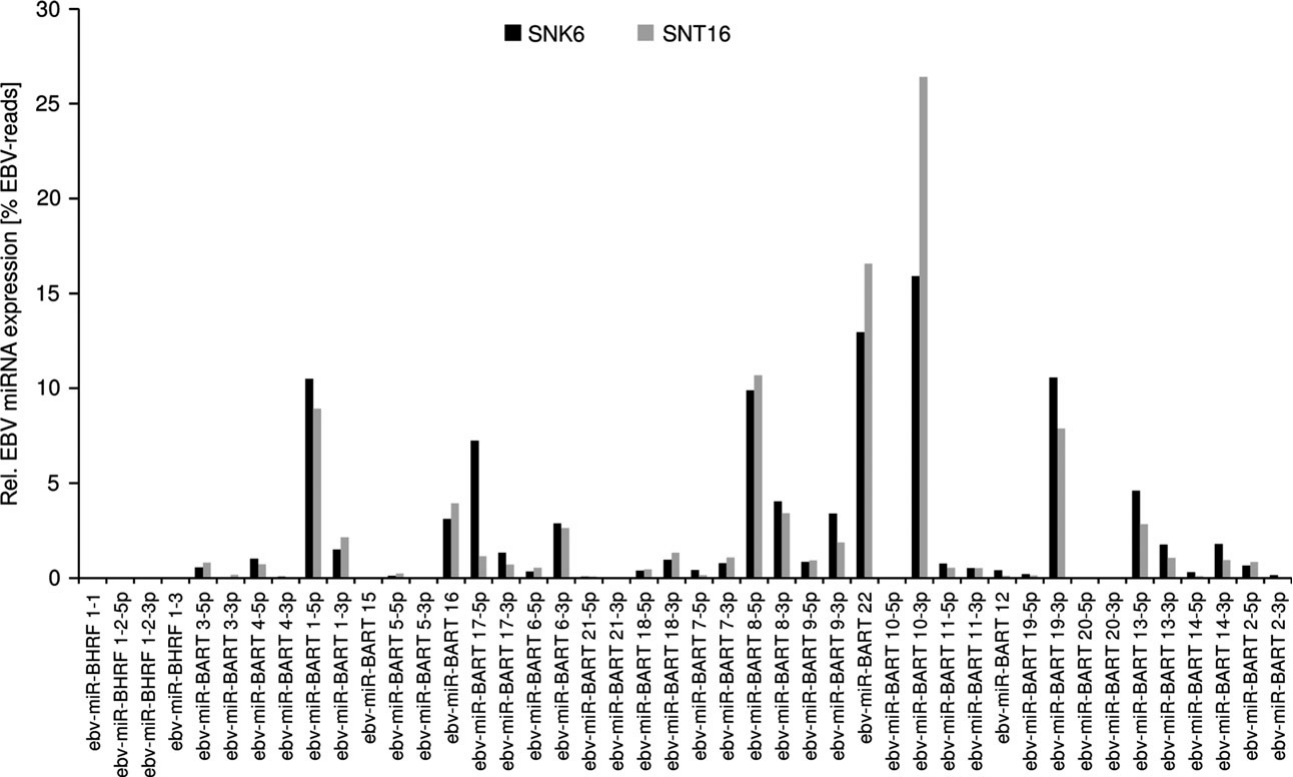
Comparison of EBV‐miRNA expression levels in NKTL‐cell lines. ebv‐miRNA expression in the SNK6 vs. SNT16 cell lines. The relative levels of the ebv‐miRNAs obtained by sequencing of the two NK/T‐lymphoma cell lines were compared with each other. Black: SNK6, grey: SNT16.

Of the EBV miRNAs, BART10, ‐22, ‐8 and ‐1 were present at the highest levels, while no BHRF1‐derived miRNAs were detectable (Fig. 1). The EBV miRNAs were compared with our previous profiling (Fig. S1). The absence of BHRF1‐derived miRNAs is consistent with prior observations that these miRNAs are only present in cells in type III latency (for review, see 16, 41). Similar results were reported for the EBV miRNAs of the two cell lines by Ling and colleagues who showed that the two lines do only marginally express the BHRF1 miRNAs 42. However, the relative levels of miRNAs detected in that publication differed from the data presented here in that we find (by sequencing) the highest expression for miRNAs BART10, ‐22, ‐8 and 19 while they report (by microarray analysis) highest expression for miRNAs BART17, ‐7, ‐1 and 16. We assume that the different methods (sequencing vs. microarray) are responsible for these discrepancies. However, both methods come to the conclusion that the BHRF1 miRNAs are only poorly expressed if at all.

**Figure 2:**
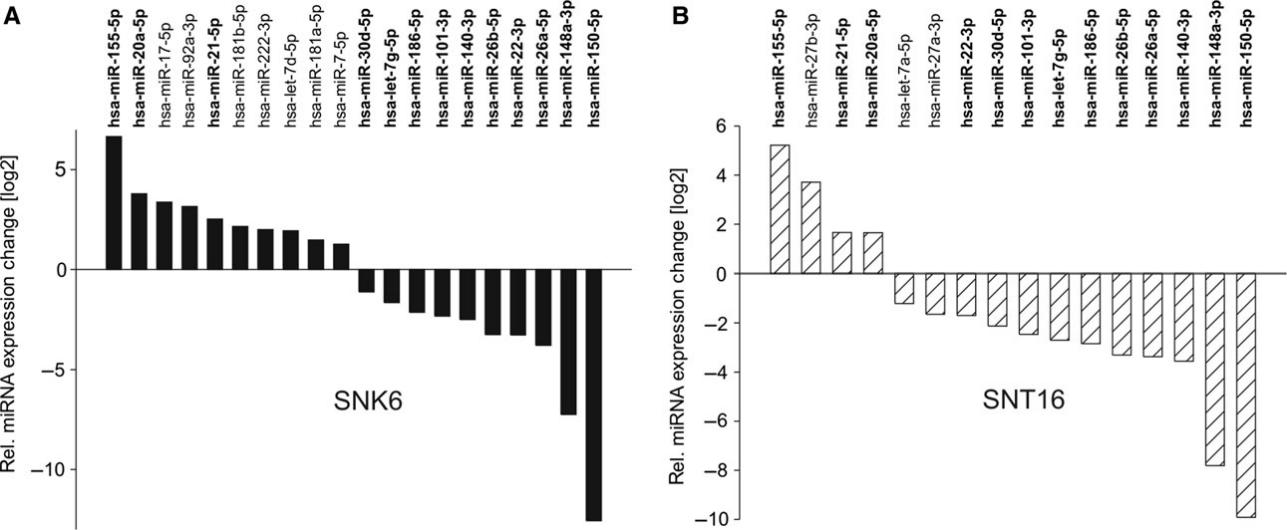
MicroRNA profiling of primary CD56+/CD3+ NK/T cells and the NK/T cell lines SNK6 (A) and SNT16 (B). MiRNAs up‐ or down‐regulated at least 1.5 fold in SNK6 or SNT16 cells compared to primary CD56+/CD3+ NK/T cells, with a representation of at least 0.1% in one of the cDNA libraries are depicted.

**Figure 3:**
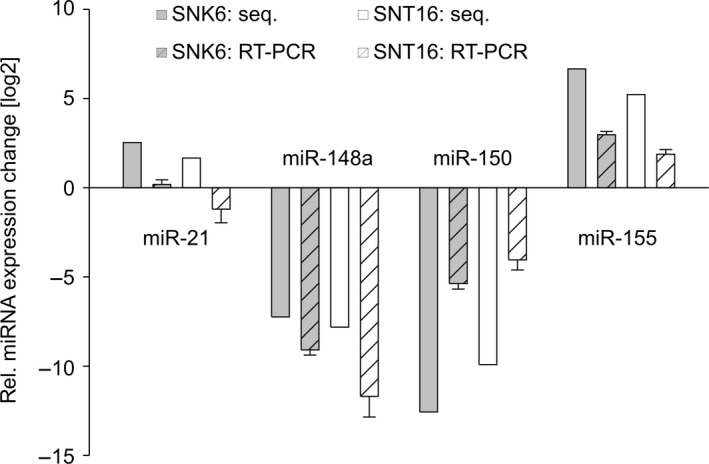
Validation of the Illumina results. The relative levels (SNK6 or SNT16/primary cells) of the four miRNAs hsa‐miR‐21 and hsa‐miR‐155 (determined as up‐regulated by sequencing) and hsa‐miR‐148a and hsa‐miR‐150 (determined as down‐regulated by sequencing), analysed by qRT‐PCR are shown. The graph represents the results of at least three independent experiments. Error bars show SD.

**Figure 4:**
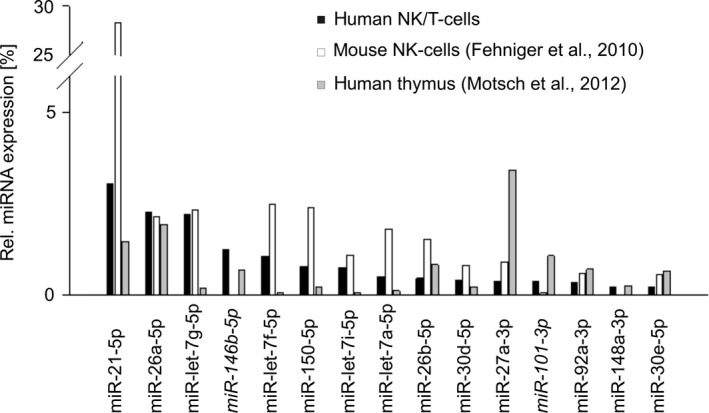
Top 10 miRNAs in human NK/T cells vs. mouse NK cells vs. thymus. Comparison of the top 10 miRNAs expressed in primary human CD56+/CD3+ NK/T cells (black) to our previous sequencing analysis of thymus tissue (grey) 33 and the sequencing analysis of activated mouse NK cells (white) 43. (comment on miRNAs in italic: miR‐146b: sequencing data from thymus counted for miR‐146a+b. miR‐101: among mmu‐miRNAs there are miR‐101a/b/c, in the human miRNA database only miR‐101‐5p and ‐3p are listed. Therefore, the sequencing data for mmu‐miR‐101 do not show up in this graph).

The relative up‐ or down‐regulation of cellular miRNAs in the SNK6/SNT16 vs. the primary CD56+/CD3+ cells was determined (Fig. 2A,B respectively). For this comparison, only miRNAs were considered that represented at least 0.1% of all reads in one of the libraries and that showed a relative change of at least 1.5 relative to the CD56+/CD3+ normal cells. In both cell lines the highest relative up‐regulation was observed for miR‐155‐5p and the strongest down‐regulation was found for miR‐150‐5p. Following the above criteria, there were 10 up‐regulated and 10 down‐regulated miRNAs in the SNK6 line, while only four miRNAs (miR‐155, ‐27b, ‐21, and ‐20a) were up‐regulated and 12 others were down‐regulated in the SNT16 line. The relative differences in the levels of miR‐21, ‐148a, 150 and ‐155 were determined by qRT‐PCR which confirmed the sequencing results for miR‐148a, ‐150 and ‐155 but not for miR‐21 (Fig. 3). miRNAs miR‐222‐3p and miR‐423‐5p were up‐regulated in the SNK6 cells but down‐regulated in SNT16. Conversely, miR‐27‐3p was down‐ in SNK6 but up‐regulated in SNT16. The relative levels of miRNAs from the present analysis were compared with two earlier studies establishing the expression profile of miRNAs in normal thymus tissue vs. primary NKTL 33, and in primary murine NK cells 43 (Fig. 4). Greater divergence is seen in the values obtained from thymus, as compared to the human primary NK/T and mouse primary NK cells, which likely reflects the fact that the thymus is composed of different cell types. The SNK6/SNT16 cell lines were also compared with the previous sequencing of primary NKTL 33. The 10 miRNAs with the highest expression levels in each entity are shown in Table 1. In the two cell lines, eight of the 10 miRNAs were identical, while only three highly expressed miRNAs from the primary tumour samples were also among the top 10 found in the SNK6/SNT16 cell lines.

**Table 1:** Top 10 miRNAs expressed in NK/T‐cell lines vs. primary NK/T‐cell lymphoma

SNK6		SNT16		NKTL 33	
miRNA	Rel. expr. (%)	miRNA	Rel. expr. (%)	miRNA	Rel. expr. (%)
hsa‐miR‐21‐5p	17.65	hsa‐miR‐21‐5p	9.67	hsa‐miR‐16	9.09
hsa‐miR‐155‐5p	7.75	hsa‐miR‐155‐5p	2.85	hsa‐miR‐23b+23a	7.23
hsa‐miR‐92a‐3p	3.08	hsa‐miR‐146b‐5p	2.24	hsa‐miR‐21	5.87
hsa‐let‐7f‐5p	1.69	hsa‐let‐7f‐5p	1.00	hsa‐miR‐27a	5.01
hsa‐miR‐146b‐5p	1.68	hsa‐miR‐27b‐3p	0.59	hsa‐miR‐26a	4.52
hsa‐let‐7i‐5p	1.05	hsa‐let‐7i‐5p	0.55	hsa‐miR‐199a‐3+199b‐3p	4.08
hsa‐let‐7 g‐5p	0.71	hsa‐miR‐92a‐3p	0.43	hsa‐miR‐27b	3.31
hsa‐miR‐20a‐5p	0.68	hsa‐let‐7 g‐5p	0.34	hsa‐miR‐15a	2.96
hsa‐let‐7a‐5p	0.52	hsa‐let‐7a‐5p	0.22	hsa‐miR‐26b	2.9
hsa‐miR‐30e‐5p	0.39	hsa‐miR‐26a‐5p	0.22	hsa‐miR‐145	2.31

### Identification of new targets for viral and deregulated host miRNAs

**Figure 5:**
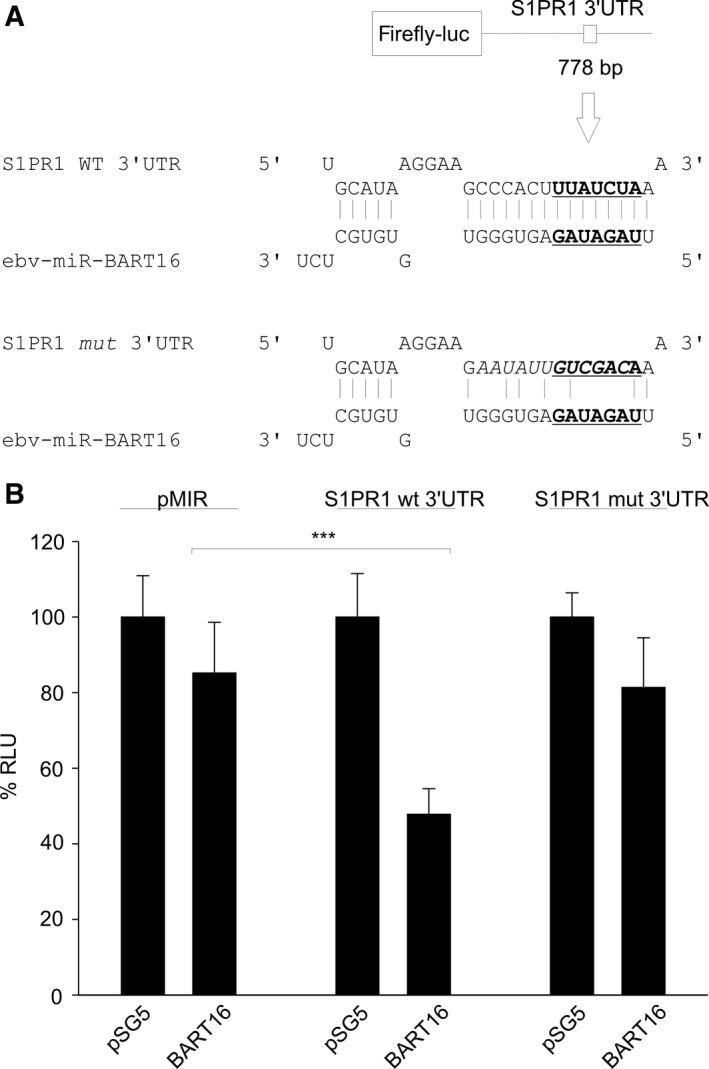
S1PR1 3′UTR is targeted by ebv‐miR‐BART16. (A) Schematic representation of the pMIR‐S1PR1 3′UTR reporter construct (upper panel) and prediction of the ebv‐BART16 binding site. The mutated binding site for BART16 is shown in the lower panel. (B) Dual‐luciferase reporter assays for ebv‐miR‐BART16 and S1PR1 3′UTR. Co‐transfection of the S1PR1 WT‐reporter construct with pSG5‐BART16 results in a significant decrease in luciferase activity compared to the empty pMIR reporter vector. Using the S1PR1 reporter with mutated BART16 binding site, no effect was observed. The graph represents the results of at least three independent experiments carried out in duplicate. Error bars show SD. Stars denote: ***P < 0.001.

A bioinformatical analysis using the ‘TargetScan Human Custom' algorithm (http://www.targetscan.org/vert_50/seedmatch.html) predicted the 3′UTR of the sphingosin‐1‐phosphate receptor 1 (S1PR1) to be a target of ebv‐miR‐BART16 (Fig. 5A). In this experiment, curiously, expression of BART16 showed reduced luciferase activity with the empty reporter vector. Nevertheless, inclusion of the S1PR1 3′UTR in the reporter down‐modulated activity to a significantly greater degree (*P* < 0.0001) and this enhanced down modulation was abrogated by mutation of the potential binding site, demonstrating its specificity (Fig. 5B). We have recently shown that coexpression of the EBV‐encoded EBER RNAs co‐represses certain targets such as down modulation of IL‐1α by miR‐142‐3p but had no effect on the regulation of TOMM22 by ebv‐miR‐BART16 28. Expression of EBER1/2 also down modulated the empty vector and coexpression with BART16 increased this. Significantly, EBER1/2 cooperated with BART16 in the specific down modulation of the S1PR1 3′ UTR (*P* = 0.002). In conjunction with the previous report showing that BART16 had no effect on TOMM22 28, this again illustrates that the effect of the EBERs are specific for a given miRNA vis‐à‐vis its mRNA target.

**Figure 6:**
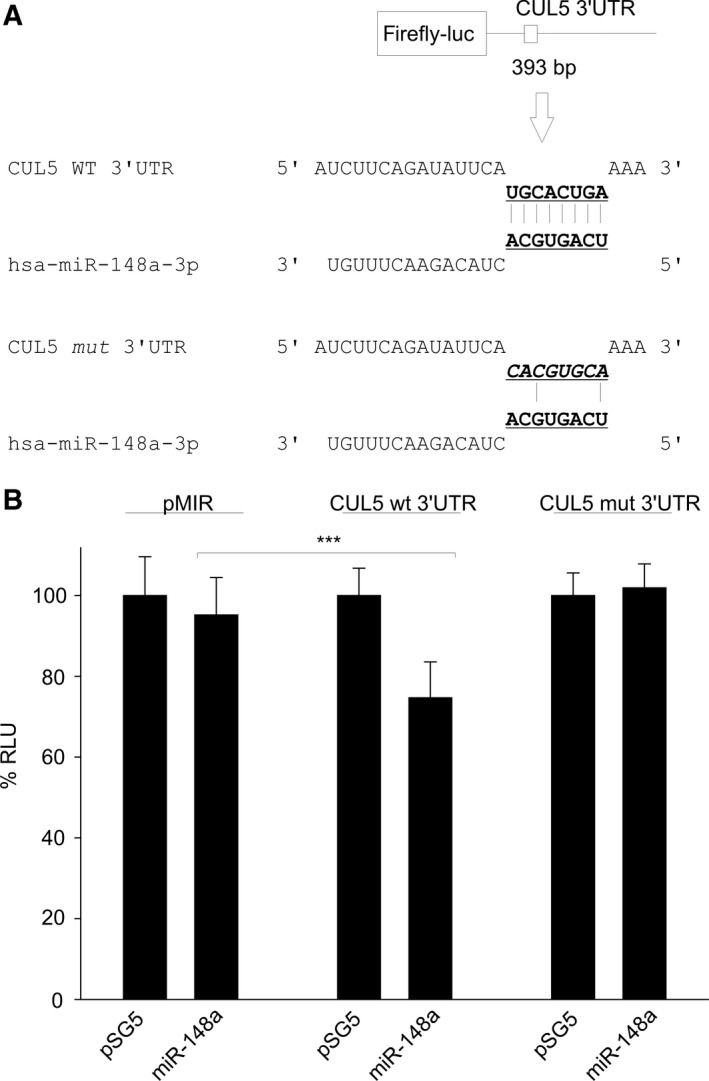
CUL5 3′UTR is targeted by hsa‐miR‐148a. (A) Schematic representation of the pMIR‐CUL5 3′UTR reporter construct (upper panel) and prediction of the miR‐148a binding site. The mutated binding site for miR‐148a is shown in the lower panel. (B) Dual‐luciferase reporter assays for miR‐148a and CUL5 3′UTR. Co‐transfection of the CUL5 WT‐reporter construct with pSG5‐miR‐148a results in a significant decrease in luciferase activity compared to the empty pMIR reporter vector. Using the CUL5 reporter with mutated miR‐148a binding site, no effect was observed. The graph represents the results of at least three independent experiments carried out in duplicate. Error bars show SD. Stars denote: ***P = 0.0005.

Using the miRecords database (http://c1.accurascience.com/miRecords/prediction_query.php), a combination of 11 miRNA target prediction programs, an interaction for the strongly down‐regulated hsa‐miR‐148a with the cullin 5 (CUL5) 3′UTR was predicted in 6 out of 11 algorithms (Fig. 6A). In reporter assays, miR‐148a overexpression resulted in a significant decrease in the relative luciferase activity using the wt‐CUL5 reporter vector (*P* = 0.0005), but this effect was abrogated when the CUL5 reporter with the mutation of the potential binding site was cotransfected. Coexpression of the EBERs had no additional effect (data not shown). Successful expression of ebv‐miR‐BART16 and hsa‐miR‐148a in 293T cells used in the luciferase assays was verified by northern blotting (Fig. S2).

**Figure 7:**
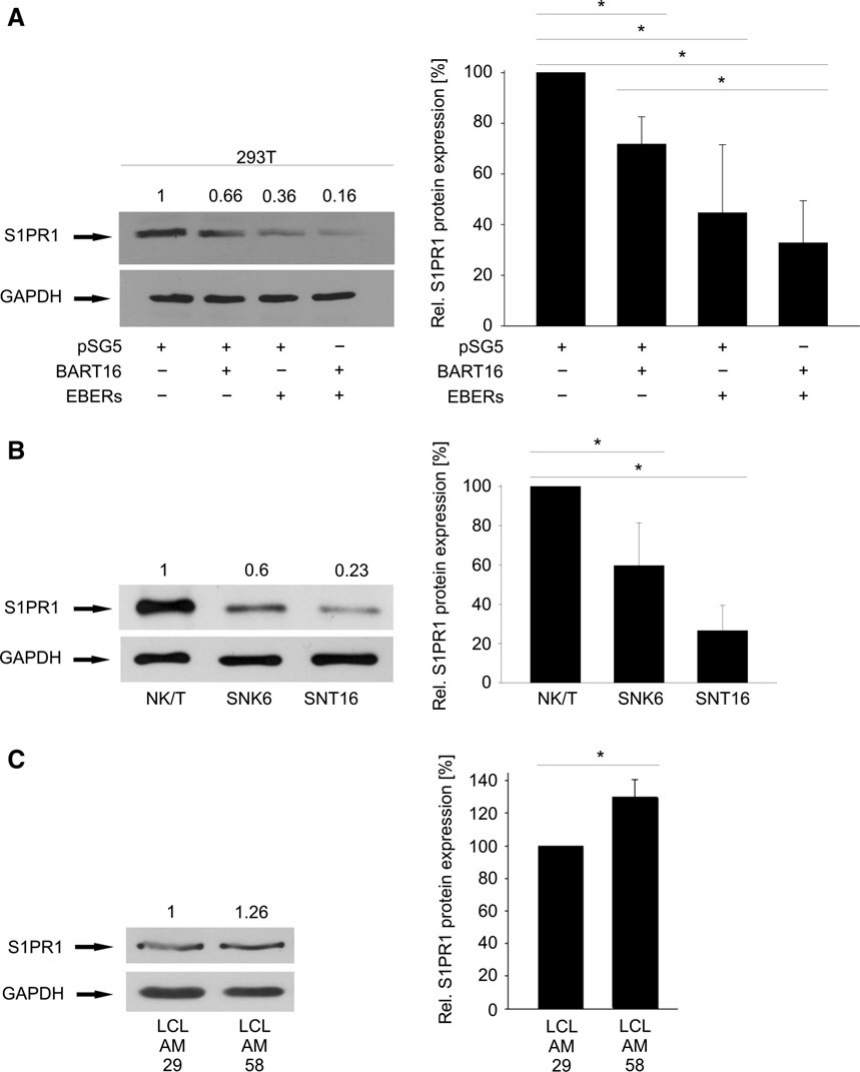
Down‐regulation of S1PR1 protein by ebv‐miR‐BART16 and EBER. Western Blot analyses of S1PR1 levels in cell lines. (A) The empty pSG5 vector, pSG5‐BART16 and pSG5‐EBERs were transfected in HEK293T cells. The expression of BART16 and EBERs alone down‐regulated S1PR1 levels significantly (*P = 0.01, *P = 0.02 respectively). Co‐expression of BART16 and the EBERs further down‐regulated the S1PR1 protein level to a stronger extent than the BART16 plasmid alone (*P = 0.03) (B) S1PR1 is down‐regulated in NK/T‐cell lines (SNK6 and SNT16) compared to nontransformed CD56+/CD3+ primary cells (*P = 0.03, *P = 0.0006 respectively). (C) S1PR1 levels in lymphoblastoid cell lines established with an EBER‐deleted EBV (LCL AM 58) or with the parental EBV (LCL AM 29) (*P = 0.008). The quantification of at least three independent experiments is shown on the right side of a representative blot. Error bars show SD.

As BART16 exerted a significant effect on the luciferase reporter in the absence of the S1PR1 3′UTR the activity of BART16, with and without the EBERs, on S1PR1 protein levels was assayed directly. Overexpression of BART16 (*P* = 0.01) or the EBERs (*P* = 0.02) in HEK293T cells resulted in a down modulation of the S1PR1 protein and the effect was additive with expression of both BART16 and the EBERs (*P* = 0.03) (Fig. 7A). We then compared the S1PR1 expression levels in the EBV‐infected SNK6/SNT16 NK/T cells with primary CD56+/CD3+ NK/T cells isolated from healthy blood donors. The levels of endogenous S1PR1 were significantly lower in the EBV‐transformed SNK6 and SNT16 cells compared to primary NK/T cells (*P* = 0.03 and 0.0006 respectively) suggesting that S1PR1 levels may be down modulated by EBV‐transformation (Fig. 7B). Successful expression of ebv‐miR‐BART16 and EBER1/2 in 293T cells used in the western blots was verified by northern blotting. Furthermore, no up‐regulation of BART16 by the EBERs was observed, eliminating the potential of a higher ebv‐miR‐BART16 level causing stronger S1PR1 protein down‐regulation in this experiment; in fact, the BART16 levels were slightly lower in the EBER/BART16 co‐transfection arguing against the effect of induced BART16 due to the presence of the EBERs (Fig. S3). In summary, our data establish S1PR1 as a novel target for BART16. To further corroborate these results, we compared the S1PR1 protein levels in two LCL lines that were established using the wt‐EBV (‘LCL AM 29') and the EBER‐ knock‐out virus (‘LCL AM 58') 34. Here, the AM 58 cell line with the EBER deletion had higher S1PR1 levels than the EBER‐positive AM 29 cells (*P* = 0.008) (Fig. 7D).

**Figure 8:**
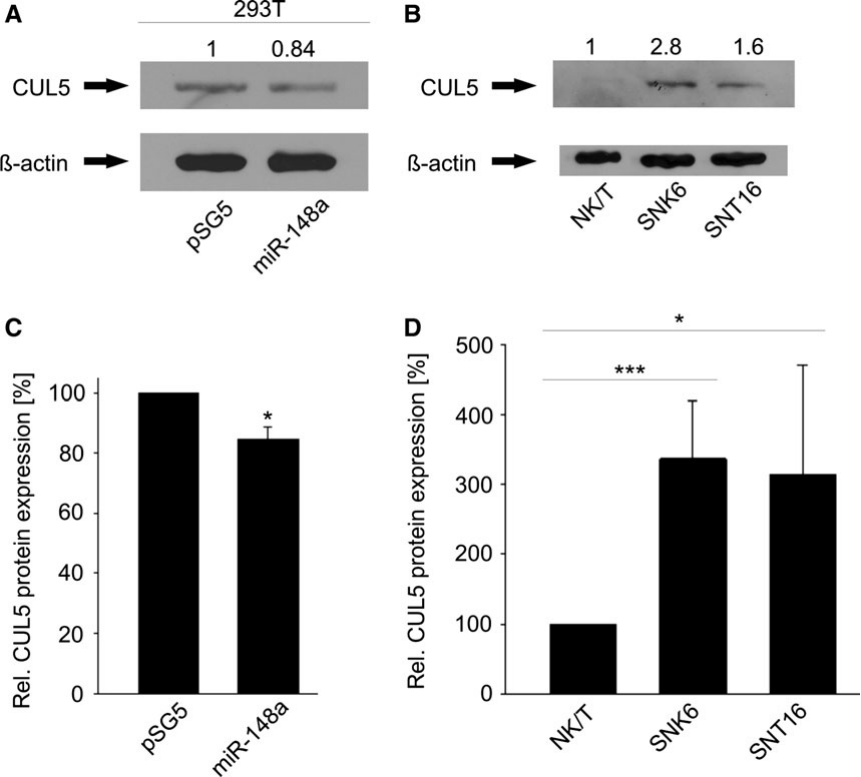
Down‐regulation of CUL5 protein by hsa‐miR‐148a. Western Blot analyses of CUL5 levels. (A) pSG5‐miR‐148a and the empty control (pSG5) were transfected in HEK293T cells. miR‐148a overexpression down‐regulated the CUL5 level significantly (*P = 0.0003). (B) CUL5 is up‐regulated in NK/T‐cell lines (SNK6 and SNT16) compared to nontransformed CD56+/CD3+‐primary cells (***P = 0.0002, *P = 0.016 respectively). (C, D) Western Blot quantification of at least three independent experiments. Error bars show SD.

By virtue of the positive luciferase reporter assays for miR‐148a and CUL5, we further investigated the CUL5 protein levels as a consequence of miR‐148a expression (Fig. 8A/C). Transfection of the miR‐148a expression plasmid resulted in a significant reduction in the CUL5 protein level in HEK293T cells (*P* = 0.0003). We then further analysed the endogenous CUL5 protein levels in the EBV‐positive SNK6/SNT16 cells compared to primary CD56+/CD3+ NK/T cells. In the NKTL cell lines, in which miR‐148a expression was strongly down‐regulated, the CUL5 protein level showed an induction of about threefold (*P* = 0.0002 and 0.016 respectively) (Fig. 8B/D). Together, these data establish S1PR1 and CUL5 to be the new targets for ebv‐miR‐BART16 and hsa‐miR‐148a respectively.

## Discussion

Here, we present miRNA profiling of the cell lines SNK6 and SNT16 derived from peripheral NKTL. The up‐ or down‐regulated cellular miRNAs were largely similar in the two cell lines compared to the CD56+/CD3+ primary NK/T cells. However, we observed differences when comparing these data with results obtained in a prior miRNA profiling carried out with primary NKTL 33. The discrepancies in the absolute values in the cell lines vs. tumour tissue may primarily be explained by the fact that the tumour contains stromal tissue. MiR‐205, which was found to be down‐regulated in the prior analysis, was strongly up‐regulated in the SNK6/SNT16 cell lines compared to the CD56+/CD3+ primary cells. Comparison of the CD56+/CD3+ primary cell data with the profiling of primary murine NK cells 43 showed good concordance. The sequencing of the SNK6/SNT16 cell lines (which are in latency type II, LMP1‐positive but no expression of EBNA2 and EBNA3s) revealed the virtual absence of BHRF1‐derived miRNAs confirming previous observations (reviewed in 16, 41). A recent report described the down‐regulation of miR‐15a in primary nasal NKTL 44. We confirmed the down‐regulation of miR‐15a, ‐223, ‐150 and ‐342 in our analysis. It was also reported that ebv‐miR‐BART20‐5p down‐regulated T‐bet (TBX21) in nasal NKTL 45. Here, as previously 33, we found only relatively low levels of ebv‐miR‐BART20‐5p. However, in the SNK6 and SNT16 cell lines analysed, the EBV‐encoded miRNAs overall represented 31.4% and 14.7% of all miRNAs reads consistent with the notion that EBV‐encoded miRNAs play a pivotal role in tumorigenic transformation by EBV 46. The difference in expression of, i.e. hsa‐miR‐223‐3p, ‐423‐5p or ‐27‐3p, might be due to the fact that SNK6, which is CD3‐negative 35 has features of NK cells while SNT16 is presumably of T‐cell origin 36.

S1PR1 (also called EDG1), a new target regulated by ebv‐miR‐BART16, was originally found in differentiating human endothelial cells 47 and represents one of five sphingosin‐1‐phosphate (SIP)‐activated G‐coupled receptors (reviewed in 48, 49). SIP inhibits cell migration during endothelial to mesenchymal transformation in cardiac development 50. In addition to other cell types, S1PR1 is expressed in cells of lymphoid origin and has now been designated as CD363 51, 52. Depending on the cell type and co‐expression of the other S1P‐receptors, S1PR1 may either inhibit or activate cell growth and /or cell motility (reviewed in 53). Down‐regulation of S1PR1 expression was found in breast carcinoma 54 and low levels of S1PR1 were correlated with higher proliferation of glioma cells and are a predictor for poor survival of glioblastoma patients 55. Down‐regulation of S1PR1 was observed in lung carcinoma as compared to normal lung tissue, and the metastatic potential of a lung cancer cell line was correlated with lower S1PR1 levels 56. In contrast, chronic activation of S1PR1 by S1P, the product of sphingosine kinase 1 (SphK1) in colitis‐associated cancer increases the risk for colon cancer 57. Strong up‐regulation of S1PR1 was observed in 40/40 mantle cell lymphoma, while the same report found S1PR1 expression only in 2/37 DLBCLs 58. Inhibition of S1PR1 retarded DLBCL cell growth via reduction of STAT3 *in vitro* 59. In Hodgkin's lymphoma, 7/56 cases showed staining for S1PR1. Finally, it was demonstrated that S1PR1 and the chemokine receptor CX3CR1 are down‐regulated in nonsmall cell lung tumour‐derived NK cells, while CXCR5 and CXCR6 were induced 60. Our results support the notion that down‐regulation of S1PR1 may contribute to tumour formation in NKTL. However, this issue has to be resolved by determination of S1PR1 levels in primary NKTL tissue which was beyond the scope of this work.

Another novel target in this study validated for the strongly repressed host miR‐148a was CUL5 (also known as VACM1). As a member of 7 Cul‐RING‐E3 ubiquitin ligases (CRLs), CRLs, in combination with different substrate receptor proteins, facilitate the conjugation of ubiquitin residues to target proteins which then were designated to degradation by the proteasome. As their role in cancer and other diseases is dependent on their substrate receptors and target proteins, cullins are nontypical tumour suppressors or oncoproteins. Many publications suggested CUL5 to function as a tumour suppressor, for example, through inhibition of Src‐dependent tumorigenesis 61. As a target for miR‐19a/b, CUL5 induces proliferation and invasion in cervical cancer cells 62. Inhibition by miR‐7 triggers G1/S transition in hepatocellular carcinoma cells 63. Mediating Jak3 degradation together with CUL1 as a consequence of NOTCH activation, CUL5 affects B‐ and T‐cell development 64. In addition, several viral substrate receptors for CUL5 have been reported: the HIV Vif protein causes CUL5‐dependent degradation of the antiviral APOBEC3G 65, 66. The Adenovirus 5 E4orf6 and E1B55K collaborate to target the tumour suppressor p53 for degradation 67. A similar mechanism is known for the KSHV LANA, which promotes proteasomal degradation of p53 and VHL in KSHV‐infected tumour cells 68. In EBV‐infected cells, CUL5 catalyses p53 poly‐ubiquitination due to its binding by the viral substrate receptor BZLF1 69. BZLF1 triggers EBV to switch from latent to lytic cycle replication. EBV typically is in a latent type of infection but EBV needs to reactivate and replicate to some extent. Our data suggest that an up‐regulation of CUL5 not necessarily maintains tumour growth rather than EBV transmission and spread within NKTL tissues.

A recent study from the Cullen group revealed that the percentage of a miRNA expressed in cells does not necessarily indicate its impact on post‐transcriptional target gene repression as the amount of RISC‐associated miRNAs better reflects their potential of down‐regulating targets 70. According to that result, the loading of the RISC complex with miRNAs will be an important additional experiment to determine functional relevance. Furthermore, a recent publication showed that the majority of miRNAs in primary, resting cells are localized in nonfunctional, low‐molecular weight complexes while the biologically active, mRNA‐associated miRNAs are preferentially found in high‐molecular weight complexes 71. Our results do not include the analysis of RISC association, however, the consequence of our newly confirmed targets for one viral and one strongly down‐regulated host miRNA can clearly be seen by the determination of the endogenous level of those proteins in the nontransformed primary CD56+/CD3+ NK/T cells compared to our two EBV‐positive lymphoma cell lines.

## Conclusions

The high‐throughput sequencing of the two NKTL cell lines revealed that the miRNA profiling yields a largely different set of deregulated miRNAs as compared to the profiles obtained when primary NKTL tissues are examined. The observation that the viral miRNAs represent a large fraction of the total miRNA pool implies a relevant contribution to the induction and/or maintenance of the transformed phenotype. In line with previous observations of EBV‐positive tumour tissues, the cell lines do not express the EBV‐encoded BHRF1 miRNAs. We show that the highly deregulated miRNA hsa‐miR‐148a down‐regulates cullin 5 (CUL5) and that the viral miRNA ebv‐miR‐BART16 down‐regulates the sphingosin‐1‐phosphate receptor 1 (S1PR1). The potential importance of the down‐regulation of S1PR1 is highlighted by the fact that the EBV‐encoded EBER RNAs exert a co‐repression on the S1PR1 protein levels. We again show that the co‐repression by the EBERs is specific for a given miRNA as we observe no co‐repression by the EBERs on CUL5 and hsa‐miR‐148a or on TOMM22, a previously established target for ebv‐miR‐BART16.

## Author contributions

G.M. and F.A.G. planned experiments; J.A., N.M., M.H., N.E., R.R., performed experiments and/or analysed data; J.A. and F.A.G wrote the paper.

## Supporting information


**Fig. S1.** Comparison of EBV miRNA expression levels.

**Fig. S2.** Ectopic expression of ebv‐miR‐BART16 and hsa‐miR‐148a in 293T cells.

**Fig. S3.** Transfection control of EBER1, EBER2 and ebv‐miR‐BART16 in 293T cells.

**Table S1.** Sequencing data (EBV miRNAs) reads obtained and relative ebv‐ miRNA expression in SNK6/SNT16 cDNA libraries.

**Table S2.** Sequencing data (host miRNAs) Reads obtained and relative hsa‐miRNA expression in NK/T and SNK6/SNT16 cDNA libraries.
