# Supplementary figures and images for: miRNA expression profiling of Epstein–Barr virus‐associated NKTL cell lines by Illumina deep sequencing
Source: FEBS Open Bio. 2016 Feb 27;6(4):251–63. doi: 10.1002/2211-5463.12027 (PMC4821355; doi:10.1002/2211-5463.12027)

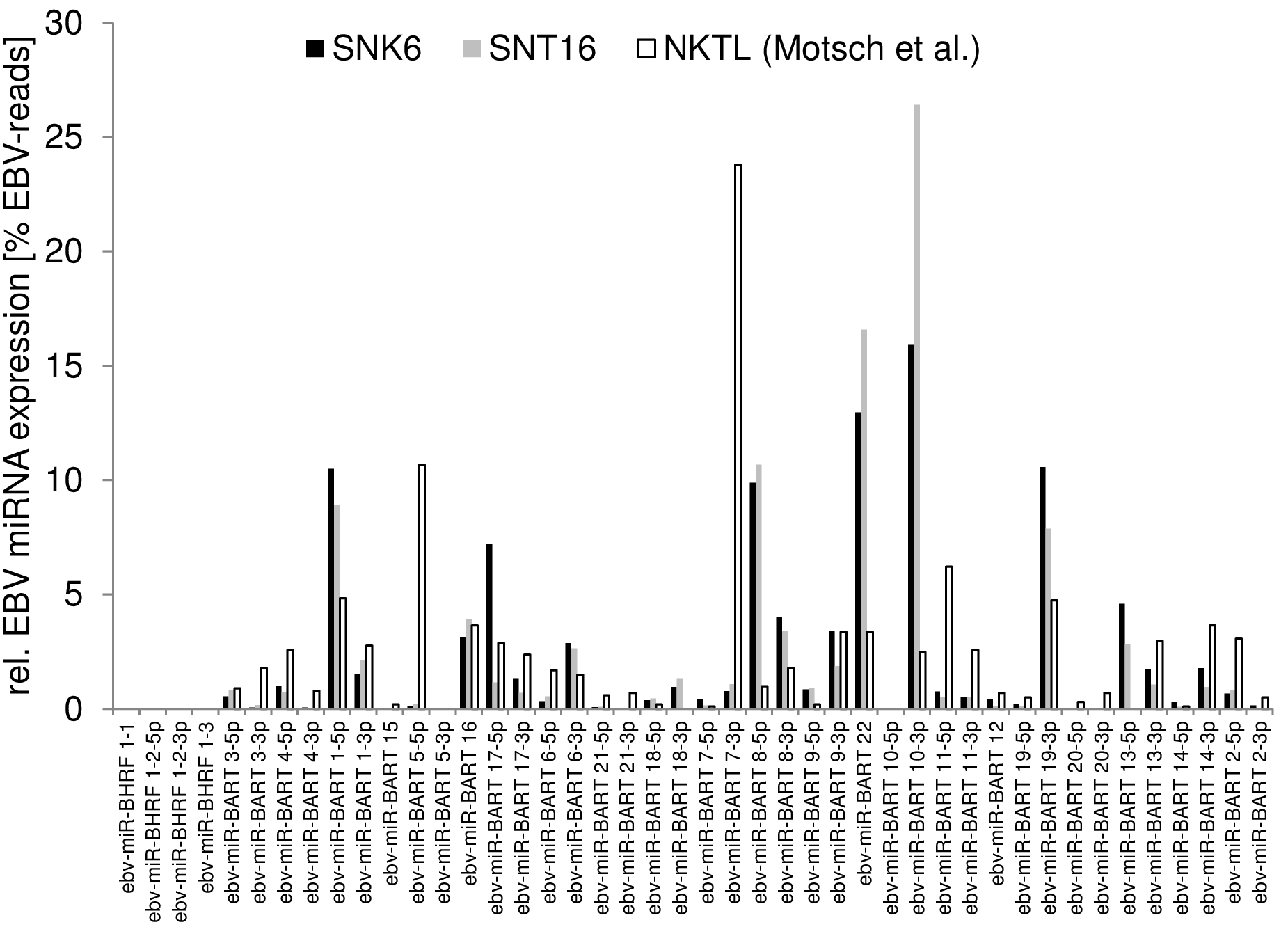

Supplement: Supplementary file 1 — Fig. S1. Comparison of EBV miRNA expression levels. [file FEB4-6-251-s001.tiff]

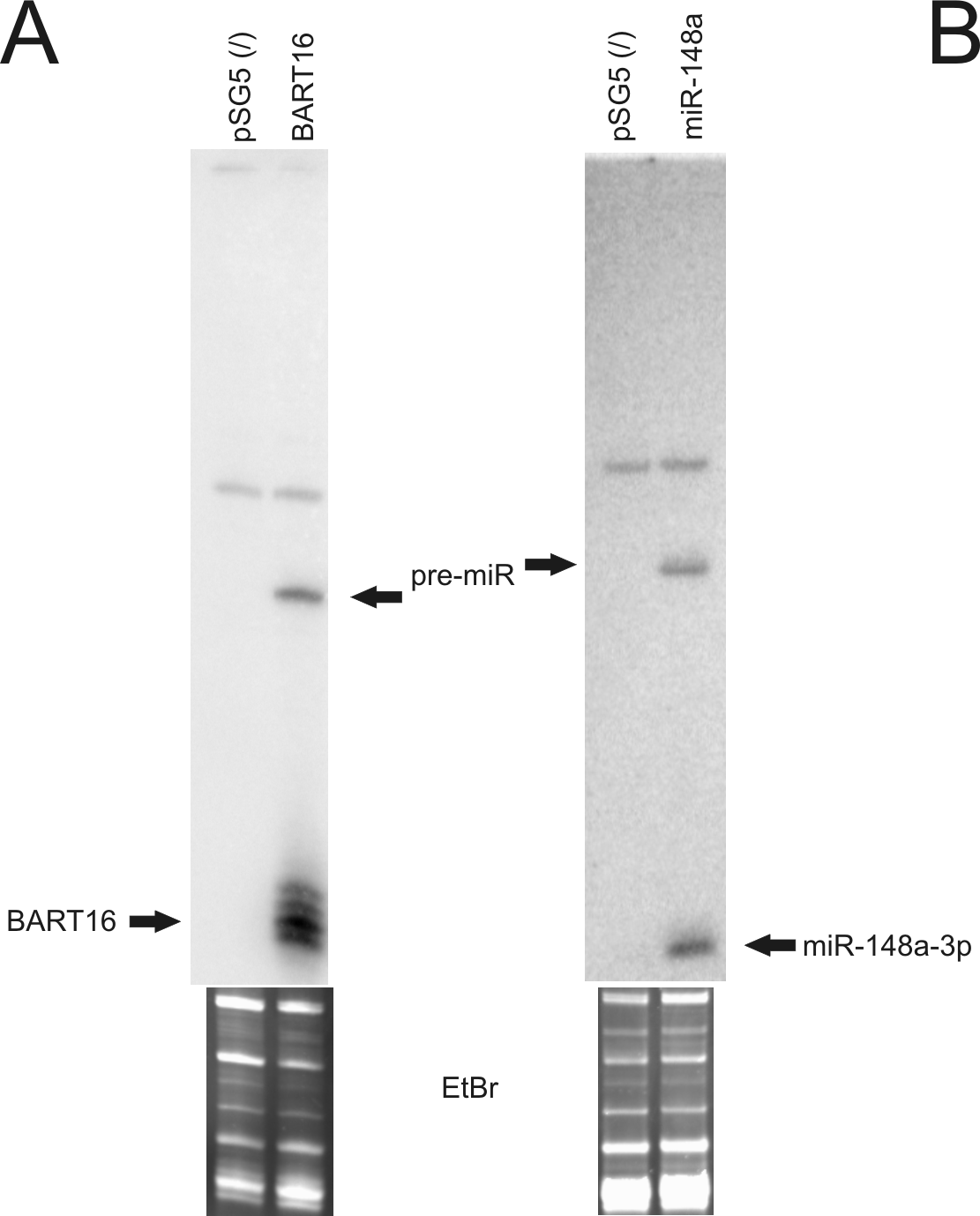

Supplement: Supplementary file 2 — Fig. S2. Ectopic expression of ebv‐miR‐BART16 and hsa‐miR‐148a in 293T cells. [file FEB4-6-251-s002.tif]

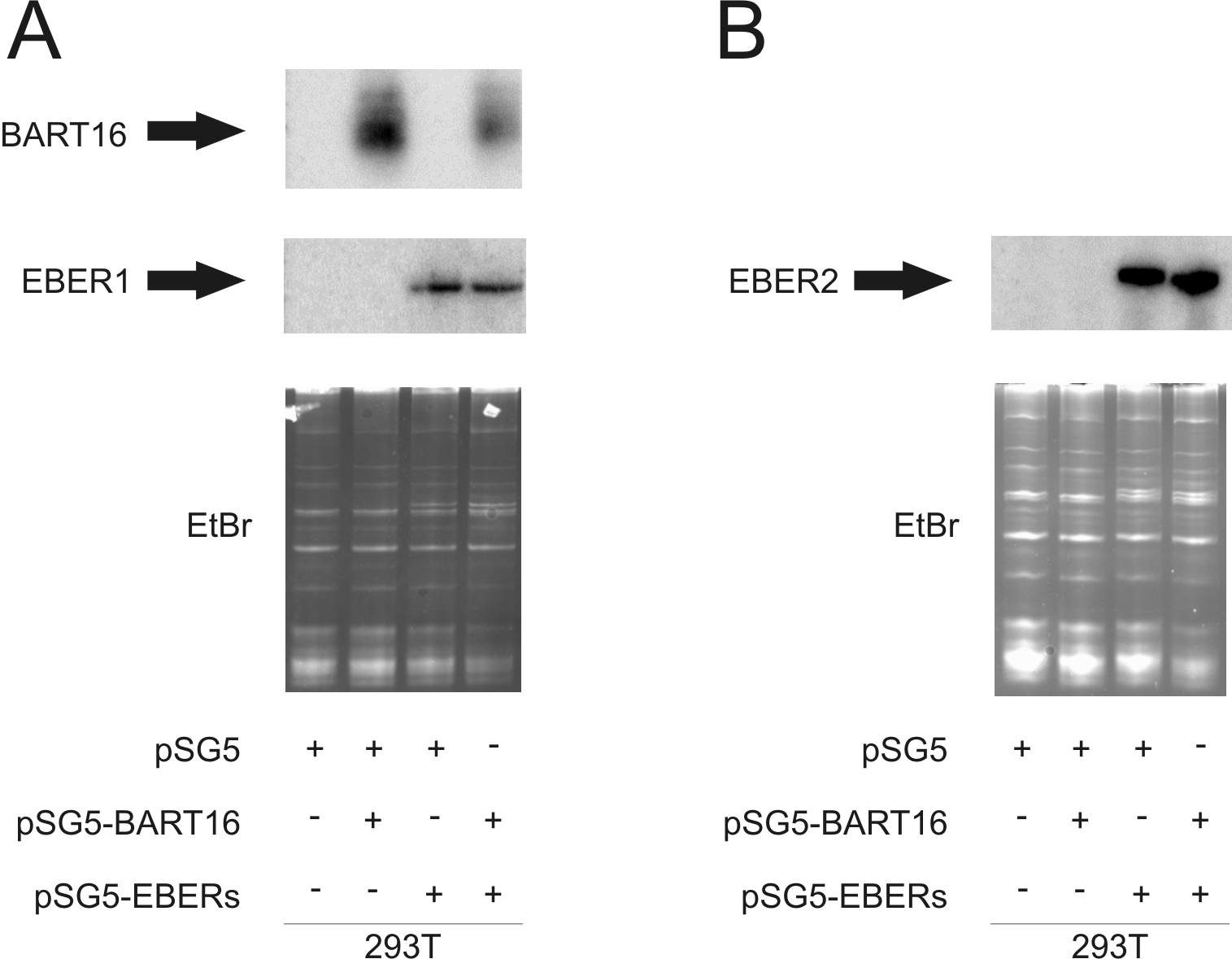

Supplement: Supplementary file 3 — Fig. S3. Transfection control of EBER1, EBER2 and ebv‐miR‐BART16 in 293T cells. [file FEB4-6-251-s003.tif]
